# Unnecessary Hysterectomies Among Premenopausal Women in Developed and Developing Countries: A Critical Review of Steps Taken to Improve Women's Health

**DOI:** 10.7759/cureus.49943

**Published:** 2023-12-05

**Authors:** Hitaishi Aggarwal, Hardik Aggarwal, Anil Wanjari

**Affiliations:** 1 Medicine, Jawaharlal Nehru Medical College, Datta Meghe Institute of Higher Education and Research, Wardha, IND; 2 Medicine, All India Institute of Medical Sciences, Rishikesh, Rishikesh, IND

**Keywords:** premenopausal women, sociopsychological health, health education, fibroids, hysterectomy

## Abstract

Women with pelvic organ disease often require a hysterectomy for better health. Still, in countries like India, there are many challenges for them as they are subjected many a time to unnecessary hysterectomies. Through this article, we suggest many ways to address this menace, such as proper health education and sensitization of women of premenopausal age group by health workers and mass media. Many preventable hysterectomies can be avoided if we guide women about their pathology before giving consent for surgery. Role enhancement of health agencies and nongovernmental organizations for early identification of such women in the society of all segments, stopping the greed of various health institutions using government-sponsored insurance health schemes for personal gain, and law enforcement by courts and health departments should also be our main focus. This study aims to review nonsurgical procedures adopted to decrease unnecessary hysterectomies and make suitable directions by government and lawful agencies to curb this menace. All states and union territories received the "Guidelines to Prevent Unnecessary Hysterectomies" recommendations from the Ministry of Health and Family Welfare for compliance. The recommendations suggest creating hysterectomy monitoring committees at the district, state, and federal levels. The duration of hospital stays and associated expenditures can decrease by performing minimally invasive treatments as outpatient operations. Minimally invasive options may shape the future of gynecology in developed nations.

## Introduction and background

Women or their relatives must discuss all alternative treatment options before adopting extreme choices such as hysterectomies for fibroid, abnormal uterine bleeding, prolapse, chronic lower back pain, pelvic inflammatory disease, abnormal uterine lining thickening (endometrial hyperplasia), and premalignant, malignant uterine, and cervical tumors. Many nonsurgical, low-cost interventions are recommended to improve the health of women in the reproductive age group. The cost of menstrual care and hygiene in some contractual sets is also a triggering factor with the aim of more work hours for women in such settings. Private institutions are misusing health insurance money for personal gains by misguiding women from poor social and economic backgrounds such as scheduled castes, scheduled tribes, and backward communities.

## Review

Search methodology

**Figure 1:**
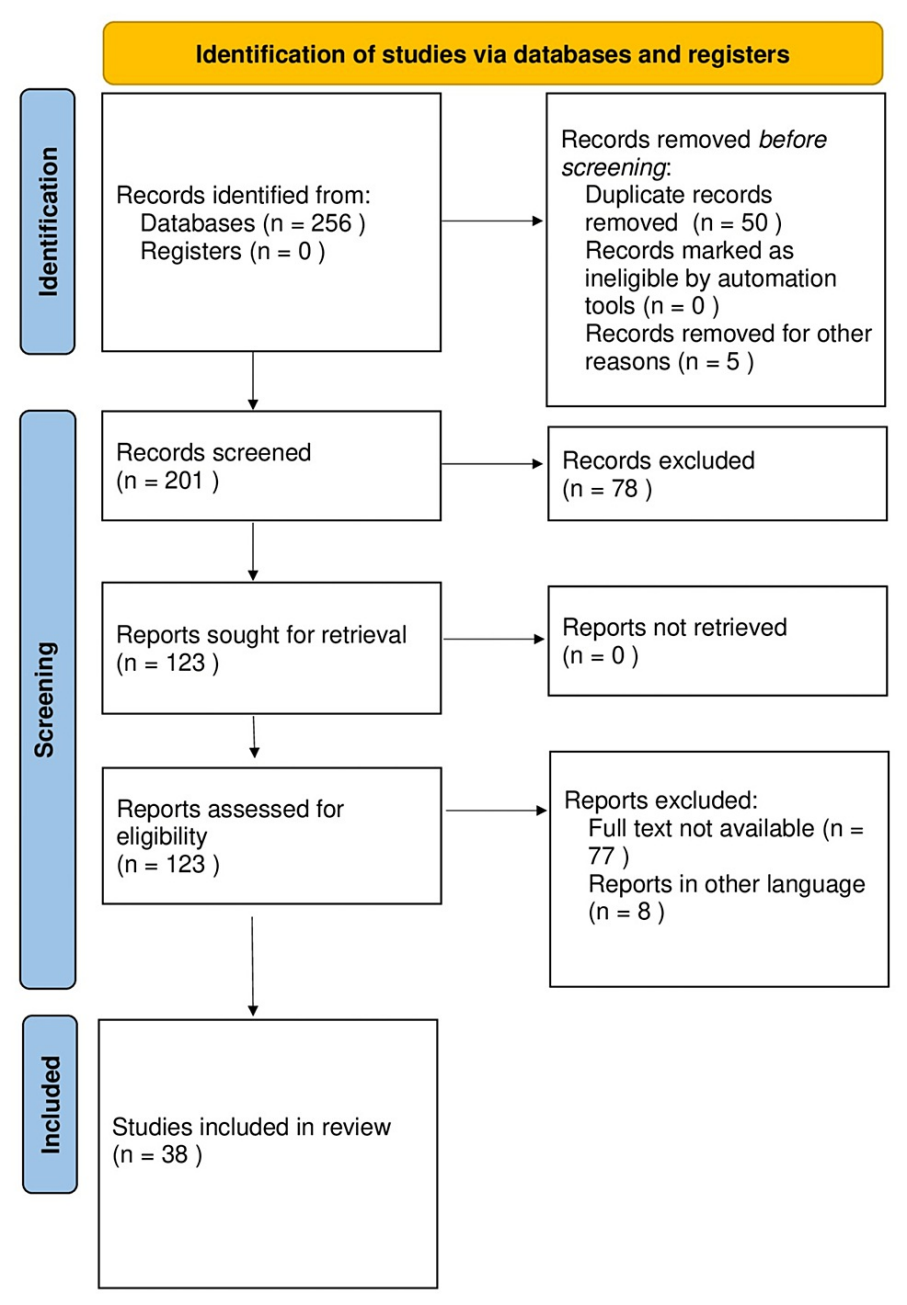
Screening and the number of articles included in the final review

Embase, PubMed, Cumulative Index of Nursing and Allied Health Literature, SINOMED, Cochrane Library, Ovid MEDLINE, and Web of Science databases were rigorously searched and reviewed from 1974 and updated until June 2023 by two authors. Research and reviews published in English during the last more than 50 years were considered in this review. In earlier years, less emphasis was framed regarding women's health and indications for hysterectomy, but in due course of time, more guidelines and emphasis are framed to improve the health of women of the reproductive age group. Various complications due to unnecessary hysterectomies, such as sexuality, pain, psychological well-being, early onset menopause, etc., and their controversial views and empirical data are debated. Particular emphasis is placed on the design, sample, measurement difficulties, and several nonsurgical approaches to hysterectomy, their early detection, and the role of nongovernmental organizations, health departments, and law enforcers critically compared using keywords such as "health education," "socio-psychological health," "fibroids," "pre-menopausal women," and "hysterectomy" (("health education" [Title/Abstract]) OR ("health education" [MeSH Terms]), ("Socio-psychological health" [Title/Abstract])) OR (("Socio-psychological health" [MeSH Terms]), ("Fibroids" [MeSH Terms]), AND ("Pre-menopausal women" [Title/Abstract])) OR ("Pre-menopausal women" [MeSH Terms]) (Figure 1).

Results

In the 1974 research by Richards, 70% of patients who underwent hysterectomies experienced postoperative depression, and almost as many also experienced hot flashes, urinary problems, and acute fatigue. The majority of these symptoms are due to oophorectomy [1]. In 1989, a study showed that individuals undergoing hysterectomy had considerably higher psychological morbidity (60%) [2]. In 1993, 42 hospitals in Pennsylvania provided data for a study on 4,660 patients, indicating that doctors in various institutions had distinct philosophies regarding practicing medicine. Many hospitals have fewer validating signs for large volumes of hysterectomies where obstetrics and gynecology programs are being run [3]. During a 2001 review of 63 insurance claims and semi-structured interviews with 12 providers by members of the Self Employed Women's Association, an Indian Central Bureau of Health Intelligence scheme, in Gujarat, operating rooms were found to lack separate handwashing facilities or proper lighting, and there were no qualified nursing staff. Hysterectomies were also conducted on demand, and removed organs were not submitted for histology even when symptoms and indications point to an illness [4]. In the United States, between 1994 and 1999, 52% of all hysterectomies were performed in women under 44 years. Three diagnoses accounted for 73% of all hysterectomies: uterine leiomyoma, endometriosis, and uterine prolapse. Bilateral oophorectomies performed concurrently with laparoscopically assisted vaginal hysterectomies dramatically increased from 20.4% in 1994 to 42.5% in 1999 [5].

In 2005, authors discovered that 10%-20% of patients with hysterectomy for benign causes experience adverse psychosocial consequences such as decreased sexual desire, altered body image, and worsened depressive symptoms [6]. The psychological effects of a recent hysterectomy for non-malignant diseases were investigated prospectively in 68 women [7]. Forty-four young women who had undergone hysterectomies for various reasons, such as fear of cancer, failure of medical treatments, difficulties coping with reproductive health concerns, and terror instilled by doctors, were interviewed in-depth in 2010 with the conviction that surgery was the best option [8]. A longitudinal prospective cohort study addressing ovarian failure due to hysterectomy for benign diseases and annual follow-up with serum follicle-stimulating hormone levels in all women less than or equal to 46 concluded that early ovarian failure developed in those women having unilateral oophorectomy or vaginal hysterectomy [9]. A systematic review and meta-analyses measured the relationships between the age of menarche, education level, parity, and hysterectomy for uterine fibroids and endometriosis.

In contrast to urban women who utilize the public sector more frequently, rural women use the private sector [10]. According to a 2011 research by the non-governmental organization Life-Health Reinforcement Group, Andhra Pradesh has one of the lowest median ages for women having hysterectomies internationally at 29 years due to the state's high prevalence of hysterectomies [11]. Research conducted in Gujarat by the Self Employed Women's Association, a group that aids rural women, discovered that women who underwent hysterectomies were under 35 years old [12]. According to a 2013 study, numerous groups in Indian society are violating people for poverty and illiteracy, especially rural women. The need for insurance, gender bias, and unethical behavior in the medical industry were all highlighted by these unneeded hysterectomies in the Indian area of Medak [13]. Women with less education and poor working conditions have common risk factors for poor health outcomes and diabetes and cardiovascular disease. A survey in India in 2011 among 2,214 rural and 1,641 urban women from the low-income category in Ahmedabad showed that more rural (9.8%) than urban (5.3%) women had hysterectomies among the insured category, which is more than the uninsured category [14].

In 2014, in Maharashtra, interviews with 44 women younger than 45 who had undergone hysterectomies revealed that the factors that led them to accept hysterectomy were fear of cancer, disappointment with medical outcomes, and the challenges of living with reproductive health issues. Private hospitals performed the majority of these hysterectomies to earn quick money [15]. Audit measures are essential to ensure judicious hysterectomies [16,17]. Doctors frequently promote hysterectomy as a preferable therapeutic choice for reproductive health issues; nevertheless, research suggests that this procedure has a detrimental impact on women's physical health and compromises their ability to work [18]. Health education at gynecological clinics before opting for a hysterectomy will help maintain their self-esteem and good body image [19]. Health campaigners and civil society organizations have urged that hysterectomies be included during the fourth cycle of the National Family Health Survey and that the data gathered be utilized to develop recommendations for performing uterine removal procedures [20]. In India, we need to address the flaws in healthcare governance at the community level by empowering women regarding their health and perception [21]. In Gujarat, India, a study in 2017 suggested that health policy initiatives must increase reach to women's reproductive as well as sexual healthcare, education, research, and audits to encourage women to make excellent decisions throughout the life cycle [22].

The emergence of ulipristal acetate as a medical etiological therapy has significantly modified clinical indications as a less invasive surgical method and reduced potential hazards and pointless procedures [23]. More education and information on the possible adverse effects of hysterectomy with additional alternatives can help women make more educated decisions [24]. The Comparing Options for Management: Patient-centered REsults for Uterine Fibroids (COMPARE-UF) registry in 2018 compared various uterine fibroids treatments (hysterectomy, myomectomy, endometrial ablation, radiofrequency fibroid ablation, uterine artery embolization, magnetic resonance imaging-guided focused ultrasound, or implantation of a progestin-releasing intrauterine device) regarding long-lasting symptom alleviation, risk of recurrence, medical consequences, improved quality of life and sexuality, age at menopause, and fertility and pregnancy outcomes so that patients and their doctors may better comprehend the benefits and drawbacks of various treatment choices and make more educated decisions [25].

Myomectomy continues to be the gold standard treatment for fibroids, and many women have improved their quality of life [26]. Public health programs must pay greater attention to teaching women about reproductive health and try to spread it through local language media, radio, and television [27]. We now have a variety of alternatives to traditional surgery, including medical and less-invasive procedural management, with good evidence showing that gonadotropin hormone-releasing hormone agonists and progesterone receptor modulators reduce fibroid volume and improve the quality of life of women with fibroids who intend to get pregnant in the future. The United Arab Emirates has also demonstrated reduced uterine fibroid volume with better quality of life [28]. Based on the National Family Health Survey 4 univariate, bivariate, and multivariate analyses done in 2020, the frequency of hysterectomies has increased women's reproductive, sociopsychological, and physical health sufferings. Women need to be persuaded to choose high-quality prophylactic measures and alternative therapies to avoid unindicated hysterectomies [28,29]. In India, community-based studies and media critiques propose a surge in young women adopting hysterectomies in a shorter period, which has led to accusations of bad manners and heated discussions about its potential to harm younger women's health. It is highly challenging to change medical practice once a procedure has established the "standard of care." Furthermore, it is impossible to overlook the attractive reimbursement for surgery that medical professionals and institutions receive (particularly for robot-assisted surgeries). Another critical element is the lack of informed consent before hysterectomy. The consent forms for gynecological surgery are frequently open-ended, allowing doctors to remove any organ without pathology. Gynecological societies assert these treatments so nobody can criticize them for such endorsements. Common observations in the literature are shown in Table 1.

**Table 1:** Common observations in the literature NGOs, nongovernmental organizations This table is created by the author(s).

Component	Developed countries	Developing countries like India	Remarks
Literacy	Mostly educated	Mostly underprivileged/women workers	Women's health education through media, NGOs, etc. required
Informed consent before surgery	Properly informed consent followed	Informed consent mostly missing	To be strictly followed and all options of treatment be discussed beforehand
Trained physician	Available	Private sector predominance	More participation by the public sector required
Menstrual hygiene	Sensitive	Mostly reason for unnecessary hysterectomies	Prompt women's health education in all sections of life
Psychosocial complications	Depression, lethargy	Depression, sexuality, self-image	Women's health education
Availability of alternative to hysterectomy	The latest nonsurgical options are more available	Least offered, possibly due to medical insurance claims	Medical treatment options must be encouraged as in developed countries
Auditing of all hysterectomies	Data monitoring better	Many records are incomplete, no data is available	Stringent law enforcers' actions required for defaulters

**Figure 2:**
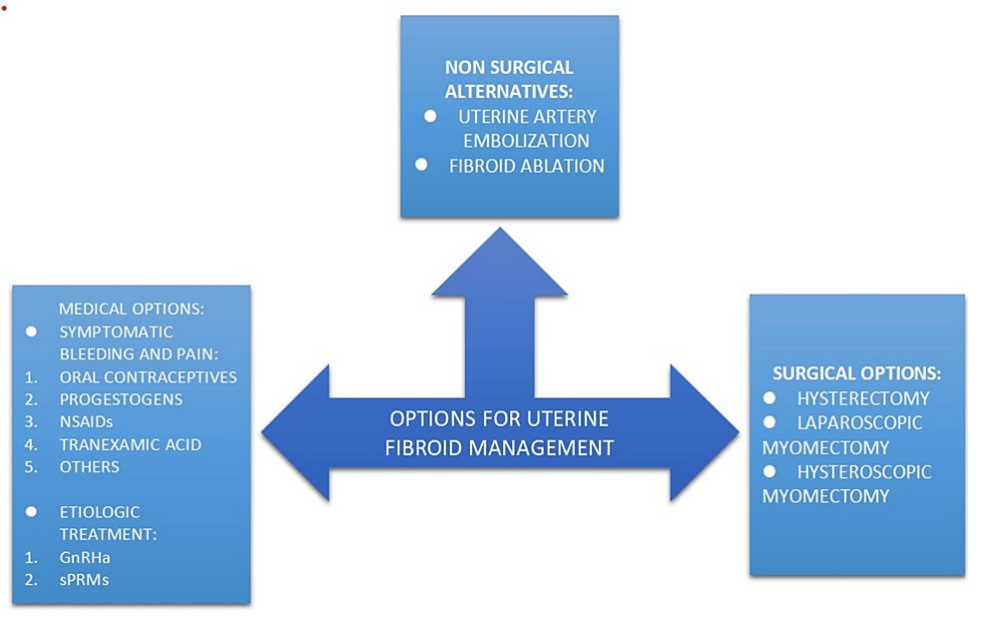
Uterine fibroid management GnRHa, gonadotropin-releasing hormone agonists; sPRMs, selective progesterone receptor modulators This figure is created by the author(s).

The average age at which hysterectomies were performed was 28.5 years, according to a study carried out in the state of Andhra Pradesh. Most private medical facilities fraudulently get exorbitant insurance premiums from the government under various health insurance programs in return. Rural India had higher rates of hysterectomy (3.4% versus 2.7%) [29]. In India, three out of every 100 women between the ages of 15 and 49 had undergone a hysterectomy, according to the results of the National Family Health Survey 5 study [30]. In national regions with higher hysterectomy rates, surveillance, audits, and advocating the prudent use of health insurance can be quite beneficial (Figure 2) [11].

Gonadotropin-releasing hormone agonists are usually advised for managing fibroids. They reduce estrogen levels, reducing symptoms associated with uterine fibroids such as heavy monthly flow and pelvic pain. Pinhole nonsurgical techniques are uterine fibroid embolization and uterine artery embolization (preserves fibroid or other sections of the uterus). Surgeons use imaging guidance to insert tiny catheters (thin tubes) into blood vessels leading up to the uterus, which houses the fibroids to deliver minute particles that obstruct circulation, causing the fibroid to cease growing and shrivel before being absorbed or killed by the body. Uterine fibroid embolization has a 95% success rate in decreasing fibroids and alleviating patient symptoms such as discomfort and heavy monthly flow. Radiofrequency ablation is an outpatient nonsurgical therapy for uterine fibroids with high-energy waves. Dangers include infection, bleeding, and scarring. Menopause may be achieved with ulipristal acetate therapy early while protecting the patient's quality of life in the least expensive economic and social manner in the younger age group [26].

The chief justice of India proposed hysterectomies for women under 40 only with the approval of two trained doctors. Medical audits for all unnecessary hysterectomies based on patient profiles and reasons for hysterectomies, the use of alternative and effective medical treatment before hysterectomies, ethical issues relating to surgical options, justification of surgical options, complications of the surgery, whether these surgeries are covered under health insurance schemes, blood transfusion for correcting anemia, cost of surgery, etc. should be made more effectively at district and state levels to protect women from adopting such course of treatment and putting them under distressful medical health issues such as depression and social disgrace. The Supreme Court also suggested the creation of national-, state-, and district-level hysterectomy monitoring committees, as well as the establishment of a grievance site, to address the issue [31,32].

Discussion

According to statistics from the National Family Health Survey 5, 3% of women between the ages of 15 and 49 have had a hysterectomy. The greatest prevalence of hysterectomy among women aged 15-49 is seen in Andhra Pradesh (9%), followed by Telangana (8%), Sikkim (0.8%), and Meghalaya (0.7%). With a rate of 4.2%, which was also higher than the national average, the southern region of India had the highest incidence of hysterectomy, followed by the eastern region (3.8%). Only 1.2% of it is in the northeast [33].

**Figure 3:**
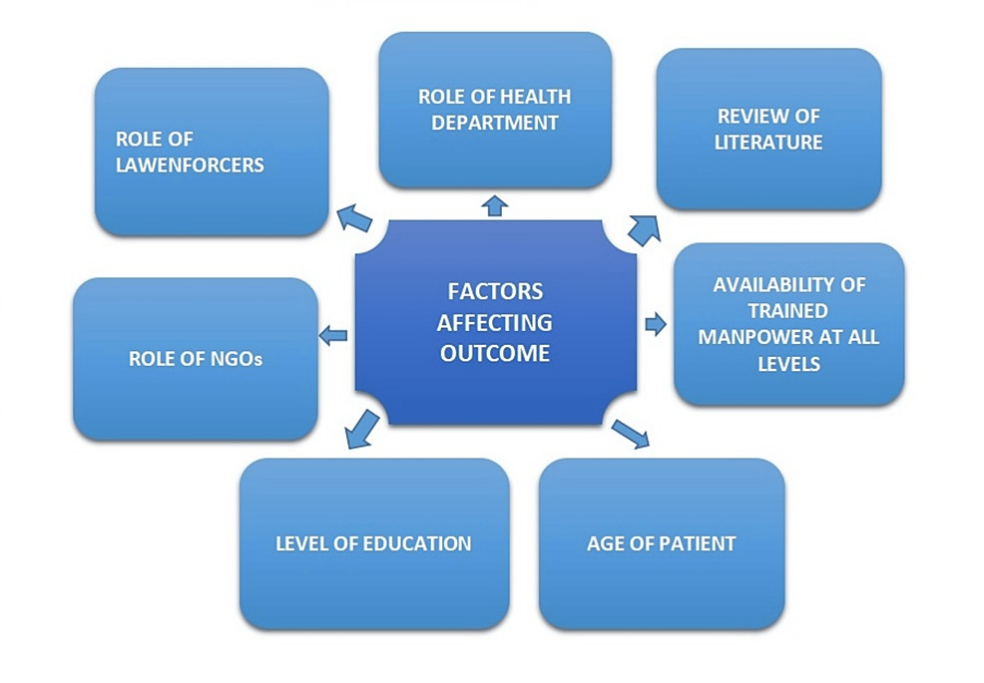
Factors affecting outcomes NGOs, nongovernmental organizations This figure is created by the author(s).

Before deciding on a hysterectomy, women must know about their reproductive health and the potential adverse effects of surgery, especially those in their early reproductive years. Their situation could improve if they are aware of nonsurgical treatment choices, and they may be able to live healthier lives for medical conditions that do not necessitate hysterectomies. To accomplish these goals, which should also reach distant areas in different sections of the nation, various resources, the mass media, public health educators, and social groups must collaborate. The most recent ruling acknowledges India's soaring hysterectomy rates among young women compared to patterns seen in affluent nations. It recognizes the violation of fundamental rights and claims that unneeded hysterectomies violate Article 21 of the Constitution's right to life and health [34]. With Rs. 5 lakh annual health coverage per family, covering 12 crore families under Ayushman Bharat Pradhan Mantri Jan Arogya Yojana, the long-term impacts of this very promising scheme must be observed to see how it would curb this danger (preventing needless hysterectomies) from society via prudent usage [35]. The factors affecting outcomes are shown in Figure 3.

**Figure 4:**
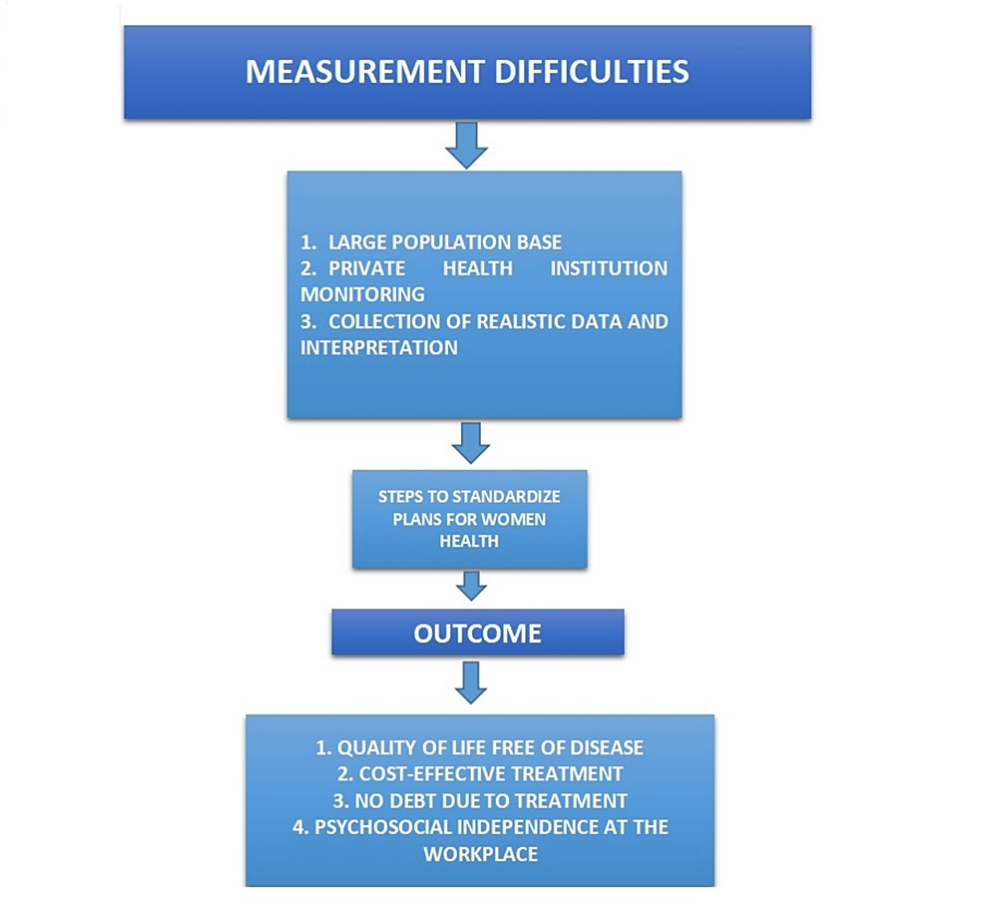
Design sample and measurement difficulties This figure is created by the author(s).

Doctors in remote areas of the country try to create fear among premenopausal women because of their less education and social shyness. They fall prey to their hands for choosing unindicated hysterectomy and subsequently suffer from various ill effects such as early menopause, early aging, psychosocial distress, cardiovascular symptoms, and decreased bone marrow density; it also sometimes affects their sexual life. Prolapse due to malnutrition and hard labor is one of the reasons for such uncalled hysterectomies. Government-sponsored insurance programs Rashtriya Swasthya Bima Yojana and Ayushman Bharat Pradhan Mantri Jan Arogya Yojana provide free treatment to families below the poverty line or other equivalent beneficiaries. The main aim of such health insurance is to reach all beneficiaries in remote areas of the country by empaneling health institutions with all health facilities with a noble motive to achieve a healthy nation. Qualified doctors are not available at many government health institutions in these remote or tribal areas, resulting in private health institutions playing a role in providing such services in these areas. There is a problem with accountability and government control in certain healthcare institutions, resulting in poor file-keeping and a lack of auditable data. Effective solutions face obstacles such as the requirement for records of all hysterectomies, external audits of bodies, and surveys of public, private, and camp environments. Establishing rules and procedures, standardizing health examinations, and utilizing online or short message service-based notifications and discharge forms could also be helpful. Informed consent from patients must convey a complete understanding of the benefits, drawbacks, and options available (Figure 4).

Preventing unnecessary hysterectomies comes with several challenges, including limited availability of two gynecologist opinions before hysterectomies under 40 years of age, regulating surgeries in younger women with benign medical indications, limiting hysterectomies to lifesaving procedures, monitoring private sector practices before using government-sponsored health insurance schemes, utilizing more of the government sector to avoid unindicated hysterectomies, and supporting noninvasive procedure through campaigns such as the Federation of Obstetric and Gynaecological Societies of India's "Save the uterus" initiative launched in 2019 [36]. In recent years, the number of hysterectomies for benign gynecological diseases has decreased in Australia, the United States, and Sweden. Australia saw a 25% decrease from 2002 to 2014, the United States decreased by 54.2% from 2002 to 2012, and Sweden decreased from 232 per 100,000 person-years in 1999 to around 210 per 100,000 person-years in 2003 [37]. A summary table of the studies included in the review is shown in Table 2.

**Table 2:** Summary table of the studies GHQ, General Health Questionnaire; LH, luteinizing hormone; SEWA, Self Employed Women's Association

Author name	Year of the study	Summary of the publication
Richards [1]	1974	The findings demonstrated that compared to other surgeries, hysterectomy is more frequently followed by a number of distressing symptoms.
Vyas et al. [2]	1989	Patients having hysterectomy were shown to have higher GHQ scores at the time of hospital release, as well as considerably greater rates of mental morbidity (60%) and psychiatric distress.
Carlson et al. [3]	1993	Hysterectomy is the most common major procedure in the United States, second only to cesarean sections.
Ranson et al. [4]	2001	SEWA must adopt a strategic role in healthcare procurement to raise the standard of care that is made available to the members.
Flory et al. [6]	2005	In terms of sexuality, pain, and psychological functioning, this work critically and exhaustively evaluates the psychosocial effects of hysterectomy.
Yen et al. [7]	2008	This study showed that while sexual function declined following hysterectomy, depression, body self-image, distress, and other gynecological symptoms improved.
Sardeshpande [8]	2014	This study is based on a qualitative study done in the state of Maharashtra that aims to comprehend how women get around obstacles to receiving care for reproductive morbidities, the justifications for choosing hysterectomy as "the" treatment, and the effects of hysterectomy on women's health.
Read et al. [9]	2010	Women who underwent vaginal hysterectomy or unilateral oophorectomy experienced earlier ovarian failure.
Desai et al. [10]	2011	In this study (2010), in Ahmedabad, Gujarat, India, hysterectomy prevalence among the 2,214 rural and 1,641 urban insured and uninsured women and low-income groups was studied.
Singh et al. [11]	2021	In this study, major stress is put on reproductive rights, informed consent, and education before subjecting them to hysterectomy.
Shekhar et al. [12]	2019	Women in their 40s and 50s and those without a high school diploma, who are obese, with high parity, with early marriage, and living in India's southern and eastern states have a higher percentage and likelihood of having a hysterectomy.
Bhushan et al. [13]	2013	This article talks about women who were already poor and were being robbed of their bodies and lives by devious processes in society through the exploitation of health insurance programs.
Gupta [14]	2013	How to restore lost or deleted ovarian function is the subject of the study.
Sardeshpande [18]	2015	Hysterectomy is frequently promoted by doctors as a preferable course of therapy for diseases of the reproductive system; nevertheless, the study reveals that this procedure affects women's health adversely and reduces their ability to work.
Yaman et al. [19]	2015	This study showed that body image and, subsequently, self-esteem are protected when patients get health education before surgery (hysterectomy).
Desai et al. [21]	2017	To quantify incidence and identify determinants of hysterectomy in a low-income context in Gujarat, India, this work used a mixed methods approach.
Acharya [22]	2017	The existing scenario surrounding needless hysterectomies in India can be improved by addressing the flaws in healthcare governance at the community level and by empowering women personally with regard to their health.
Mas et al. [23]	2017	It is important to achieve the patient's objectives and decisions by enhancing clinical diagnosis for these types of illnesses, enabling better personalized therapies, and eliminating possible dangers and unneeded procedures.
Prusty et al. [24]	2018	Better information and education about the potential side effects of hysterectomy and other options will empower women to make better educated decisions because they are also more likely to have the procedure.
Stewart et al. [25]	2018	To help patients and their providers better understand the benefits and drawbacks of various treatment choices and to help them make more educated decisions, this registry offers optimized evidence.
Fortin et al. [26]	2018	For afflicted women and society at large, uterine fibroids are a major burden. Fibroids have a significant detrimental influence on the economy, work productivity, sexuality, self-esteem, relationships, and social, emotional, and physical well-being.
Stewart et al. [27]	2019	Overall, there is strong data showing that gonadotropin-releasing hormone agonists cause a decrease in fibroid volume and an increase in a woman's quality of life.
Davis [28]	2019	This brings attention to the issue of growing hysterectomy rates in India and the dearth of comprehensive care for women suffering from the incapacitating condition of chronic pelvic discomfort.
Desai et al. [29]	2019	Hysterectomy prevalence among Indian women between 15 and 49 years of age shows the urgent need to guarantee access to treatment for major gynecological problems and avoid unnecessary hysterectomies in young women.
Meher et al. [30]	2020	Promoting high-quality preventative and therapeutic options for women is crucial as opposed to permanent but potentially harmful fixes such as hysterectomy.
Kumari et al. [31]	2022	The study reveals that two-thirds of all hysterectomies were performed in private healthcare centers in India. This study stresses the use of nonsurgical or conservative approaches as a form of gynecological morbidity treatment by special training for primary care professionals.
Li et al. [32]	2022	This study carefully analyzed qualitative information on the actual experiences of women having hysterectomies, highlighting three themes: thorough planning before the procedure, feelings and experiences following the procedure, and coping mechanisms.
Lin et al. [37]	2021	A paradigm shift for hysterectomy is evident from the major changes in surgical patterns of LH usage during the past 20 years.

## Conclusions

The availability of proper health knowledge, regular awareness through mass media, and local leaders' active participation by health system professionals is a need of the time. For women from underserved groups and cultures to get better medical advice about their issues and choose alternatives to hysterectomy, we need to strengthen the public health system. More trained and dedicated health staff and doctors are required to cover remote, underprivileged areas. Considering this burning issue on women's health, the recently worthy Supreme Court of India has lately directed all states through the Government of India health department to formulate a policy to track all hysterectomies and the reasons for their occurrence, particularly in premenopausal age groups, and define the role of gynecologists before hysterectomies. Collecting data from public and private institutions is always challenging, as they retain records poorly. Stricter laws can only make these institutions responsible for retaining such records for future reference and evaluation.
